# Connection Between Perceived Resilience and Work-Related Psychological Wellbeing Among Anglican Clergy in Wales: A Pilot Study

**DOI:** 10.1007/s10943-026-02633-2

**Published:** 2026-03-30

**Authors:** Leslie J. Francis, Ursula McKenna, V. John Payne

**Affiliations:** 1 Centre for Research in Intellectual and Developmental Disabilities (CIDD), University of Warwick, Coventry, UK; 2 World Religions and Education Research Unit, Lincoln Bishop University, Lincoln, UK

**Keywords:** Clergy studies, Burnout, Resilience, Personality, Wellbeing, Anglican

## Abstract

Drawing on data provided by a convenience sample of 123 clergy serving in parish ministry in the Church in Wales, this pilot study was designed to test the predictive power of the Brief Resilience Scale on individual differences in susceptibility to professional burnout as conceptualised and assessed by the Francis Burnout Inventory, after controlling for personal factors (sex and age) and personality factors (introversion and emotionality). The data indicated that self-perceived resilience predicted lower levels of emotional exhaustion (negative affect) and higher levels of satisfaction in ministry (positive affect). Noting the limitations of this pilot study, the case is made for additional replication studies.

## Introduction

Reviews of the ways in which the concept of resilience has been conceptualised and operationalised within the behavioural and medical sciences have drawn attention both to the increasing use of this concept and to the variety of ways in which it has been nuanced (see, for example, Ahern et al., 2006; Ayed et al., 2019; Brown et al., 2023; Carver, 1998; Charney, 2004; Fletcher & Sarkar, 2013; Masten, 2001; Pawan & Ramanathan, 2025; Rutter, 2006; Southwick et al., 2014; Tusaie & Dyer, 2004). In developing the Brief Resilience Scale, Smith et al. (2008) argued on etymological grounds that the English word ‘resilience’ is rooted in the notion of ‘bouncing or springing back’, and on scientific grounds that it is wise to distinguish this root meaning from the factors and resources that may facilitate such bouncing or springing back and from the flourish that may result from such bouncing or springing back. They conclude that:

While recognising that words evolve in meaning over time, the ability to bounce back or recover from stress may be important to assess and study in its own right. In addition this ability may be particularly important for people who are already ill or are dealing with ongoing health-related stresses. (Smith et al., 2008, p. 194)

It is this specific focus on resilience as the capacity to bounce back that distinguishes the Brief Resilience Scale from most other instruments adopting the term resilience. For example, Wagnild and Young’s (1993) Resilience Scale focused on assessing equanimity, perseverance, self-reliance, meaningfulness, and existential aloneness. Connor and Davidson’s (2003) Resilience Scale focused on assessing characteristics like self-efficacy, sense of humour, patience, optimism, and faith. Friborg et al.’s (2003) Resilience Scale for Adults focused on assessing five dimensions: personal competence, social competence, family coherence, social support, and personal structure. Sinclair and Wallston’s (2004) Brief Resilient Coping Scale focused on coping strategies like looking for creative ways to alter different situations. Rossouw and Rossouw’s (2016) Predictive Six-Factor Resilience Scale focused on assessing six domains: vision (comprising self-efficacy and goal-setting), composure (comprising emotional regulation), tenacity (comprising perseverance and hardiness), reasoning (comprising higher cognitive traits), collaboration (comprising psychological interactions), and health (comprising physiological health). On the other hand, the four-item resilience scale within the Emotional Style Questionnaire proposed by Kesebir et al. (2019) shares with the Brief Resilience Scale the specific focus on the ability to bounce back.

Smith et al. (2008) developed and tested their Brief Resilience Scale on four samples: sample one, 128 undergraduate students; sample two, 64 undergraduate students; sample three, 112 cardiac rehabilitation patients; and sample four, 50 women comparing 20 fibromyalgia patients and 30 healthy controls. The six-item measure (including three reverse coded items) recorded the following alpha coefficients: sample one, α = .84; sample two, α = .87; sample three, α = .80; sample four, α = .91. Test-related reliabilities were reported of .69 after one month among 48 participants from sample one, and of .62 after three months among 61 participants from sample three. Construct validity was established against a range of predicted correlates. The psychometric properties of the original English language form of the Brief Resilience Scale have been tested and generally supported by a number of studies, including recent studies by McKay et al. (2021) and Westcott and Rocconi (2025). Other studies, however, have proposed a two-factor solution emerging from the six items (see Tansey et al., 2016; Cabrera et al., 2023).

Since its publication in 2008, the Brief Resilience Scale has been translated into a number of languages including Arabic (Baattaiah et al., 2023), Brazilian Portuguese (Barroso, 2021; Coelho et al., 2016; da Silva-Sauer et al., 2021), Chinese (Fung, 2020; Zhang et al., 2020), Croatian **(**Budisavljevic et al., 2023), Czech and Slovak (Furstova et al., 2022), Dutch (Soer et al., 2019), French (Jacobs & Horsch, 2019; Leys et al., 2021; Todorović et al., 2025), German (Broll et al., 2024; Chmitorz et al., 2018; Kunzler et al., 2018), Greek (Kyriazos et al., 2018; Tsouvelas et al., 2022), Japanese (Limura & Taku, 2018), Korean (Kim et al., 2024), Malay (Ahmad Sabki et al., 2023), Polish (Konaszewski et al., 2020), Serbian (Despotović et al., 2025), Spanish (Hidalgo-Rasmussen & Gonzalez-Betanzos, 2019; Rodriguez-Rey et al., 2016), Turkish (Bayrak Kahraman et al., 2024; Doğan, 2015), Vietnamese (Nguyen et al., 2024), and Urdu (Khan & Batool, 2020). Cumulatively, these studies across cultures and languages have contributed to confidence in the reliability and construct validity of the Brief Resilience Scale.

### Clergy Studies

A range of recent books and academic papers has brought the notion of resilience to the forefront of clergy studies. Relevant books in the field include *Resilient pastors: The role of adversity in healing and growth* (Allain-Chapman, 2012); *Resilient ministry: What pastors told us about surviving and thriving* (Burns et al., 2013); *The resilient pastor: Ten principles for developing pastoral resilience* (Searby, 2015). Recent contributions to the academic literature include: ‘Clergy burnout and resilience: A review of the literature’ (Jackson-Jordan, 2013); ‘Resilience and professional chaplaincy: A paradigm shift in focus’ (Spidell, 2014); ‘A resilience education intervention to prevent clergy burnout’ (Abernethy et al., 2016); ‘Literature review of clergy resilience and recommendations for future research’ (Sielaff et al., 2021); ‘Resilience in Ministry: Listening to the Voice of Church of Scotland Ministers’ (McKenna, 2021); ‘(Re)Framing Resilience: A Trajectory-Based Study Involving Emerging Religious/Spiritual Leaders’ (Jankowski et al., 2023); ‘The Resilience of Clergywomen?: Gender and the Relationship between Occupational Distress and Mental Health among Congregational Leaders’ (Holleman, 2023); and a series of papers by Margaret Allison Clarke and colleagues including Clarke (2023) and Clarke et al., (2022a, 2022b, 2023).

However, only a small number of academic papers concerned with resilience among clergy have incorporated a specific measure of resilience within their reported studies. Four studies used the Connor–Davidson Resilience Scale (CD-RISC; Connor & Davidson, 2003) either in brief or full form: Noullet et al. (2018) assesses the effectiveness of pastoral crisis intervention training on resilience and compassion fatigue in 39 clergy; Baek et al. (2024) explores the factors impacting mental distress and help seeking behaviours in 110 Korean church leaders across various denomination in Los Angeles; Garcia-Rubio et al. (2025) explore the impact of stress resilience and religious coping on quality of life among 50 United Pentecostal Church of Columbia leaders; and Captari et al. (2023) explored the impact on resilience of a therapeutic intervention in a pilot study among 77 chaplains. Sohail et al. (2025) used the Emotional Styles Questionnaire (Kesebir et al., 2019) to test whether emotional styles (resilience is one of the six styles) might moderate the relationship between burnout and time in ministry among a sample of 1,027 United Methodist clergy in North Carolina, USA.

### Assessing Burnout Among Clergy

The two conceptualisations of burnout most frequently employed among clergy are the three-factor model proposed by the Maslach Burnout Inventory (MBI; Maslach & Jackson, 1986) and the two-factor model proposed by the Francis Burnout Inventory (FBI; Francis et al., 2005). There are two fundamental differences between these two models. First, the MBI conceptualises burnout in terms of sequential progression across the three factors: emotional exhaustion leads to depersonalisation, and depersonalisation leads to lack of personal accomplishment. The FBI conceptualises burnout in terms of the classic balanced affect approach to wellbeing (Bradburn, 1969): positive affect and negative affect operate as partially independent systems within which positive affect can ameliorate the detrimental consequences of negative affect. Second, while the MBI was developed for generic use among people-facing professions, the FBI was developed specifically for use among clergy, drawing on work-related experiences specifically shaped by the clerical experience. The two components of the FBI are styled the Scale of Emotional Exhaustion in Ministry (SEEM) and the Satisfaction in Ministry Scale (SIMS).

The practical insight arising from the balanced affect model of clergy burnout is that, although it may be difficult to remove from clergy experience the factors that generate negative affect, good pastoral oversight can facilitate the development of factors that support positive affect. The validity of this theory has now been established in a series of studies among 744 clergy serving in The Presbyterian Church USA (Francis et al., 2011), 155 Catholic priests serving in Italy (Francis et al., 2017c), 95 Catholic priests and 61 Catholic religious sisters serving in Italy (Francis et al., 2017a), 658 Anglican clergy serving in England (Francis et al., 2017b), 358 Anglican clergy serving in Wales (Village et al., 2018), 99 Anglican clergy serving in England (Francis et al., 2019), 287 Catholic priests serving in Italy (Francis et al., 2021), and 803 Methodist ministers serving in Great Britain (Francis et al., 2023). The present study employs the FBI.

### Taking Control Variables into Account

The consensus emerging from earlier research employing either the MBI or the FBI among clergy is that individual differences in burnout may be mapped against both personal and personality factors (for review see Francis, 2018). Of the two key personal factors (sex and age), age is the more important. In survey studies of burnout among clergy, burnout scores are routinely found to be higher among younger clergy. Two theories have been advanced to account for this routine finding: an ageing effect is reflected in older clergy being better able to manage their work-related psychological wellbeing; a cohort effect is reflected in those clergy more susceptible to burnout dropping out at a younger age.

Of the range of personality factors included in survey studies of burnout among clergy, neuroticism or emotionality routinely emerges as the strongest predictor, followed by extraversion in the second place. Emotionality and extraversion are two aspects of personality that feature across diverse models of personality including the 16 Personality Factor model (Cattell et al., 1970), the Big Five Factor model (Costa & McCrae, 1985), the Eysenckian Three Major Dimensions model (Eysenck & Eysenck, 1991), and the augmented model of psychological type theory operationalised by the Francis Psychological Type and Emotional Temperament Scales (FPTETS; Village & Francis, 2023). The present study employs the FPTETS. The personal factor of sex differences remains important in light of the established association between sex and measures of emotionality (Francis, 1993), and the factor of age differences remains important in light of the established association between age and measures of emotional exhaustion in ministry (Francis, 2018).

## Research Questions

Against this background the present study was designed to address the following three research aims:

- To test the psychometric properties of the Brief Resilience Scale among clergy
- To explore the bivariate connections among clergy between perceived resilience (as measured by the Brief Resilience Scale) and personal factors (sex and age), personality factors (introversion and emotionality), and work-related psychological wellbeing (conceptualised as positive affect and negative affect)
- To examine the predictive power of perceived resilience scores on positive and negative affect, after controlling for personal factors and psychological factors.

## Method

### Procedure

Self-supporting and stipendiary clergy serving in the Church in Wales in parish ministry were randomly invited to support a pilot study concerned with resilience in ministry. A total of 123 clergy responded and completed all the measures.

### Participants

Of the 123 participants, 71 were male, 51 female, and 1 preferred not to disclose; 20 were under the age of fifty, 35 in their fifties, 54 in their sixties, and 14 in their seventies; 85 were serving in stipendiary ministry; 7 in house for duty ministry; 11 in self-supporting ministry alongside secular employment; and 20 in self-supporting ministry without secular employment.

### Data Analysis

The data were analysed by the SPSS statistical package employing the frequency, reliability, and regression routines.

### Measure

*Resilience* was assessed by the Brief Resilience Scale (BRS; Smith et al., 2008). This six-item measure comprises three positive items (e.g. I tend to bounce back quickly after hard times) and three reverse coded items (e.g. I tend to take a long time to get over set-backs in my life). Each item is assessed on a five-point scale: agree strongly (5), agree (4), not certain (3), disagree (2), and disagree strongly (1). The four samples in the foundation study reported alpha coefficients of .80, .84, .87, and .91.

*Work-related psychological wellbeing* was assessed by the two scales proposed by the Francis Burnout Inventory (FBI; Francis et al., 2005). This 22-item instrument assesses positive affect by the Satisfaction in Ministry Scale (SIMS) and negative affect by the Scale of Emotional Exhaustion in Ministry (SEEM). Each item is assessed on a five-point scale: agree strongly (5), agree (4), not certain (3), disagree (2), and disagree strongly (1). Example items concerned with satisfaction in ministry are: ‘The ministry here gives real purpose and meaning to my life’ and ‘I gain a lot of personal satisfaction from working with people in my current ministry’. Example items concerned with emotional exhaustion are: ‘I feel drained in fulfilling my ministry roles’ and ‘I am less patient with those among whom I minister than I used to be’. In their foundation paper for the FBI, drawing on a sample of 6,680 clergy from Australia, England, and New Zealand, Francis et al. (2005) reported the following alpha coefficients: SEEM, α = .84; SIMS, α = .84.

*Introversion and emotional instability* were assessed by two scales from the Francis Psychological Type and Emotional Temperament Scales (FPTETS; Village & Francis, 2023). Each of these scales comprises ten force-choice items where the choice contrasts introversion and extraversion or contrasts emotional stability and emotional instability. The foundation paper reported mean alpha coefficients of .80 for introversion and .79 for emotional instability.

## Results and Discussion

The first step in data analysis examined the scale properties of the Brief Resilience Scale in terms of the alpha coefficients (Cronbach, 1951), the correlations between the individual scale items and the sum of the other five items, and the item endorsement in terms of the sum of the agree strongly and agree responses. The alpha coefficient of .92 identifies a tightly defined and focused construct. The correlations presented in Table 1 confirms the homogeneity of the six items with correlations ranging between .67 and .83. The highest of these correlations (.83) identifies the essence of this operationalisation of resilience as captured by the perception that ‘It does not take me long to recover from a stressful event’. The percentage endorsements indicate that between 42 and 54% agreed or agreed strongly with each of the three positively voiced items, while between 20 and 30% agreed or agreed strongly with each of the negatively voiced items.

**Table 1:** Brief resilience scale: scale properties

	*r*	%
I tend to bounce back quickly after hard times	.67	54
I have a hard time making it through stressful events (R)	.75	30
It does not take me long to recover from a stressful event	.83	50
It is hard for me to snap back when something bad happens (R)	.80	26
I usually come through difficult times with little trouble	.76	42
I tend to take a long time to get over set-backs in my life (R)	.78	20

N = 123

*r* = correlation between item and sum of the other five items

% = sum of agree and agree strongly responses

(R) = reverse coded items

The second step in data analysis explored the psychometric properties of all the scales employed by the study in terms of the alpha coefficient, the means and standard deviations. The data presented in Table 2 confirm that the Scale of Emotional Exhaustion in Ministry, the Satisfaction in Ministry Scale, the Introversion Scale, and the Emotional Instability Scale all recorded satisfactory alpha coefficients at levels consistent with previous studies.

**Table 2:** Scale properties

	N items	Alpha	Mean	SD
Brief Resilience Scale	6	.92	19.76	5.07
Scale of Emotional Exhaustion in Ministry	11	.85	27.09	7.21
Satisfaction in Ministry Scale	11	.83	43.41	4.65
Introversion Scale	10	.86	6.26	3.17
Emotional Instability Scale	10	.79	3.12	2.47

N = 123

The third step in data analysis examined the bivariate correlations among the six study variables. Two main features of these correlations merit comment (Table 3). First, scores recorded on the Brief Resilience Scale are unrelated with personal factors (age and sex) but highly related both with personality factors and with work-related psychological wellbeing. High scores of resilience are associated with extraversion and emotional stability as well as with high scores of satisfaction in ministry and low scores of emotional exhaustion in ministry. Second, personality factors are also associated with both positive affect and negative affect. Introversion and emotional instability not only predict lower sense of resilience but also lower satisfaction in ministry and higher emotional exhaustion in ministry. For these reasons it is wise now to test the association between resilience and work-related psychological wellbeing after controlling for personal and personality factors.

**Table 3:** Correlation matrix

	BRS	Sex	Age	EI	I	SIMS
SEEM	− .40^***^	− .15	− .22^*^	.49^***^	.24^**^	− .61^***^
SIMS	.40^***^	.02	.10	− .31^***^	− .32^***^	
Introversion	− .26^**^	− .13	− .06	.09		
Emotional instability	− .51^***^	− .00	− .13			
Age	.01	− .06				
Sex	− .00					

N = 123

*BRS* Brief Resilience Scale, *EI* Emotional Instability, *I* Introversion

^*^p < .05; ^**^p < .01; ^***^p < .001

Tables 4 and 5 now present three regressions models for satisfaction in ministry and emotional exhaustion in ministry respectively. In these tables personal factors (sex and age) are entered in model 1; personality factors (introversion and emotional instability) are entered in model 2; and resilience is entered in model three. In terms of satisfaction in ministry (Table 4), model 3 demonstrates that, after taking personal and personality factors into account, perceived resilience is associated with increased scores of positive affect, indicative of better work-related psychological wellbeing. In terms of emotional exhaustion in ministry (Table 5), model 3 demonstrates that, after taking personal and personality factors into account, perceived resilience is associated with decreased scores of negative affect, also indicative of better work-related psychological wellbeing.

**Table 4:** Regression models: Satisfaction in Ministry Scale

	Model 1 *β*	Model 2 *β*	Model 3 *β*
Personal factors			
Sex	.03	− .03	− .02
Age	.10	.03	.05
Personality factors			
Introversion		− .31^***^	− .25^**^
Emotional instability		− .29^***^	− .16
Resilience			
Brief Resilience Scale			.26^**^
R^2^	.011	.191	.236
Δ	.011	.180^***^	.046^**^

N = 123

^**^ *p* < .01; ^***^ *p* < .001

**Table 5:** Regression models: Scale of Emotional Exhaustion in Ministry

	Model 1 *β*	Model 2 *β*	Model 3 *β*
Personal factors			
Sex	− .16	− .12	− .13
Age	− .23^***^	− .14	− .16^*^
Personality factors			
Introversion		.18	.14
Emotional instability		.45^***^	.36^***^
Resilience			
Brief Resilience Scale			− .18^*^
R^2^	.075	.315	.337
Δ	.075^**^	.240^***^	.022^*^

N = 123

^**^ *p* < .01; ^***^ *p* < .001

### Limitations

The specific limitations with the present study include the relatively small convenience sample of 123 clergy serving in one denomination (Anglican) located in one country (Wales). A requirement of the peer-review process is acknowledgement of vulnerability to the limitation of tautology as argued by Koenig and Carey (2024), meaning the correlation of indicators of mental health with other indicators of mental health, such as satisfaction and resiliency. Naturally, these are going to be positively related, and satisfaction will be negatively related to indicators of poor mental health. The study, has, however, been successful in indicating the potential contribution of including a measure of perceived resilience within the study of individual differences in clergy work-related psychological wellbeing and professional burnout. Replication studies are now required drawing on larger samples of clergy exercising ministry within other denominations and other cultural contexts.

## Conclusion

Set within the wider context of research concerning individual differences in levels of clergy work-related psychological wellbeing and professional burnout, as conceptualised and assessed by the Francis Burnout Inventory (FBI; Francis et al., 2005), the present study was designed to assess the predictive power of perceived resilience, as conceptualised and assessed by the Brief Resilience Scale (BRS; Smith et al., 2008). This aim was progressed by addressing three sequential research question.

Since there was no previous published evidence regarding the application of the Brief Resilience Scale among clergy, the first research question examined the psychometric properties of this instrument among clergy. The data demonstrated high internal consistency reliability and confirmed the viability of proceeding the study. The high internal consistency reliability also confirmed that the BRS is a tightly defined and narrow construct.

The second research question examined the bivariate associations between perceived resilience (as measured by the Brief Resilience Scale) and personal factors (sex and age), personality factors (introversion and emotionality), and work-related psychological wellbeing (conceptualised as positive affect and negative affect). The data demonstrated that perceived resilience was positively correlated with positive affect (satisfaction in ministry) and negatively correlated with negative affect (emotional exhaustion in ministry). At the same time, the correlation matrix demonstrated the importance of personality factors in predicting individual differences both in perceived resilience and in work-related psychological wellbeing. Introversion was associated with lower perceived resilience, lower satisfaction in ministry, and higher emotional exhaustion. Emotional instability was also associated with lower perceived resilience, lower satisfaction in ministry, and higher emotional exhaustion. This finding raises the question regarding the extent to which the measure of resilience may be serving as a proxy measure for personality and confirmed the wisdom of progressing to a multivariate model of data analysis. The correlation matrix also demonstrated that personal factors (sex and age) were of less significance in the model.

Building on the first two research questions, the third research question examined the predictive power of perceived resilience on positive affect and negative affect, after controlling for personal factors (sex and age) and personality factors (introversion and emotionality). The data demonstrated that perceived resilience remained a significant predictor of higher scores on the index of satisfaction in ministry and lower scores on the index of emotional exhaustion in ministry, after controlling for individual differences in personal factors and personality factors. Two main conclusions emerge from this core finding: one theoretical and one practical.

The conclusion that contributes to theory is this. The construct assessed by the Brief Resilience Measure is more than a proxy measure for personality. This construct accessed a specific aspect of self-efficacy that is reflected in higher levels of work-related psychological wellbeing and in lower levels of professional burnout among clergy.

The conclusion that contributes to practice is this. If confidence in the ability to bounce back or recover from stress is key to impaired work-related psychological wellbeing and lower vulnerability to professional burnout, then this is a personal phenomenon worth experiencing and a skill worth developing. The next step is to develop interventions in initial and continuing ministerial formation programmes that prioritise such experience and learning.

## Data Availability

Data are available from the corresponding author upon reasonable request.
